# Parasitic Disease of Rabbits

**Published:** 1899-08

**Authors:** 

**Affiliations:** Veterinary Surgeon Bacteriologist, Melbourne Veterinary College


### PARASITIC DISEASE OF RABBITS.

By Veterinary Surgeon Desmond,
BACTERIOLOGIST, MELBOURNE VETERINARY COLLEGE.

From the Warrnambool district specimens of rabbits' livers that were thought to be affected with tuberculosis, or consumption, have been forwarded to me for examination.

The specimens were placed in diluted methylated spirit of wine, and arrived in splendid condition. They presented the following appearance: Much enlarged, paler in color than normal, easily torn, and were studded with nodules having a light-brown color, and about the size of the head of a lucifer match. When the nodules were cut across with a sharp knife a yellow discharge escaped; to a casual observer this discharge would be mistaken for pus or matter, and tuberculosis suspected. Without the aid of the microscope to one well versed in tuberculosis of animals and man it is easy to see that the well-marked conditions of this dreaded disease were absent in the specimens sent for examination. On other points tuberculosis is out of the question: A large number of rabbits are affected—this points to an enzoötic disease; while tuberculosis is prevalent in animals kept in a state of domestication, it is very rare in wild animals.

The next step was to prepare slides for microscopical examination, and for this purpose scrapings from the nodules were spread on glass slides and double-stained; on examining with the microscope the cause was found to be a small animal parasite, Coccidium oviforme.

*History.*—This parasite was first seen in the rabbit about 1830, but its true nature was for many years unknown, and has often been mistaken for the corpuscles found in pus or matter. In 1856 their true nature was discovered, and the different stages of their development fully worked out. They belong to the lowest order of animal parasites, and are known to science as protozoa. They are composed of a cell not possessing definite tissues or organs; they have the appearance of an ovoid body enclosed in a double membrane. These parasites are very small, and cannot be recognized without the aid of the microscope; they measure about the 500th of an inch in length, and about the 900th of an inch broad. There are thirteen stages in their life-history, of which six take place in the rabbit. The spores of this parasite are probably found in the earth, and are carried into the stomach with the food consumed by the animals. While in the stomach several changes take place; their next step is to migrate up the bile-ducts of the liver, which they dilate and alter the structure. After setting up a series of serious changes in the liver they are carried into the bowels and pass out of the body to complete their life-history in the soil.

Several species of coccidia are known to science, and are found in the different domesticated animals; while Coccidium oviforme is peculiar to the liver of the rabbit, it may be attacked by another coccidium which develops in the intestines. It is an exception for rabbits to be attacked by both forms of coccidia, *i.e.*, coccidium of the liver and coccidium of the intestines.

In 1883 attention was directed to a disease existing among the rabbits in Tasmania, and thought to be tuberculosis, which was destroying large numbers, and likely to be an effective means of extermination of the pest. This attracted the attention of the New South Wales Government, and a veterinary surgeon was despatched to Tasmania in December of the same year to report on the nature of the disease. As the results of the investigation, the expert in 1884 reported that the disease the rabbits were affected with was tuberculosis, and if properly worked would be the means of finally exterminating the pest in Tasmania and Australia. That the disease was not tuberculosis has since been proved; on careful investigation it was found to be coccidiosis of the liver that was mistaken for tuberculosis, an error that ought not to occur where careful microscopical examination is made. This error was wrong in two ways—from 60 to 80 per cent. of the rabbits were affected; this in itself was enough to doubt the theory of tuberculosis of animals in a wild state, while if tuberculosis could be spread among the rabbits it might be the means of making them breed faster. In my collection of specimens are several rabbits' livers from Tasmania that are affected with Coccidium oviforme, and which even to the naked eye have no resemblance to tuberculosis.

The symptoms of this disease in wild rabbits are unknown. Here is a splendid field for anyone fond of research, for which purpose I will be pleased to give all the assistance in my power to anyone who will take up the study.

It would be interesting to know if the rabbits in other parts of Victoria are affected with coccidiosis of the liver, and I would be pleased to get specimens from suspected cases.

The symptoms and treatment of domesticated rabbits affected with this disease have been worked out. As it is not legal to keep rabbits in a state of domestication in Victoria, it is not necessary to suggest a treatment. The disease in domesticated rabbits runs a slow course; it may be two or three months before death takes place. The appearances are want of condition, no appetite, fur rough and matted, dull and not timid when approached. The liver may be badly affected, and the animal show no symptoms during life of being in bad health.

There are no objections to using the animals affected with this disease as food, provided they are in good condition. The liver is the only organ that is affected, and all that is necessary is to remove it when dressing the animals.—*From the Australasian*, February 25.

